# Quantitative, real-time imaging of spreading depolarization-associated neuronal ROS production

**DOI:** 10.3389/fncel.2024.1465531

**Published:** 2024-10-11

**Authors:** Marc André Ackermann, Susanne Monika Buchholz, Katharina Dietrich, Michael Müller

**Affiliations:** Institut für Neuro-und Sinnesphysiologie, Universitätsmedizin Göttingen, Göttingen, Germany

**Keywords:** oxidative stress, reactive oxygen species, spreading depression, redox imaging, roGFP, mitochondria

## Abstract

Spreading depolarization (SD) causes a massive neuronal/glial depolarization, disturbs ionic homeostasis and deranges neuronal network function. The metabolic burden imposed by SD may also generate marked amounts of reactive oxygen species (ROS). Yet, proper optical tools are required to study this aspect with spatiotemporal detail. Therefore, we earlier generated transgenic redox indicator mice. They express in excitatory projection neurons the cytosolic redox-sensor roGFP, a reduction/oxidation sensitive green fluorescent protein which is ratiometric by excitation and responds reversibly to redox alterations. Using adult male roGFPc mice, we analyzed SD-related ROS production in CA1 *stratum pyramidale* of submerged slices. SD was induced by K^+^ microinjection, O_2_ withdrawal or mitochondrial uncoupling (FCCP). The extracellular DC potential deflection was accompanied by a spreading wavefront of roGFP oxidation, confirming marked neuronal ROS generation. Hypoxia-induced SD was preceded by a moderate oxidation, which became intensified as the DC potential deflection occurred. Upon K^+^-induced SD, roGFP oxidation slowly recovered within 10–15 min in some slices. Upon FCCP-or hypoxia-induced SD, recovery was limited. Withdrawing extracellular Ca^2+^ markedly dampened the SD-related roGFP oxidation and improved its reversibility, confirming a key-role of neuronal Ca^2+^ load in SD-related ROS generation. Neither mitochondrial uncoupling, nor inhibition of NADPH oxidase or xanthine oxidase abolished the SD-related roGFP oxidation. Therefore, ROS generation during SD involves mitochondria as well as non-mitochondrial sources. This first-time analysis of SD-related ROS dynamics became possible based on quantitative redox imaging in roGFP mice, an advanced approach, which will contribute to further decipher the molecular understanding of SD in brain pathophysiology.

## 1 Introduction

The neuronal phenomenon spreading depolarization (SD) is characterized by a massive depolarization of neurons and glial cells (Müller and Somjen, 2000), a near-complete breakdown of intra/extracellular ion homeostasis and a potentially reversible impairment of neuronal network function (Leão, 1944; Marshall, 1959; Bures et al., 1974). *In vivo*, spreading depolarization triggers a rapidly evolving reduction in the amplitudes of spontaneous activity termed spreading depression. The induction of SD follows an all-or-none principle (Somjen, 2001), and once ignited, it may spread out slowly across the gray matter in a wave-like fashion. In principle, SD may arise in most regions of the central nervous system, yet with clearly differing induction thresholds. Neocortex and hippocampus are more prone to SD than cerebellum, spinal cord, olfactory bulb or brainstem (Fifkova et al., 1961; Amemori et al., 1987; Richter et al., 2003; Funke et al., 2009). SD is triggered by various stimuli, such as increased extracellular K^+^, electrical stimulation, or hyperthermia. A generally recognized crucial component in the process of SD induction is the challenge of the Na^+^/K^+^ ATPase. This becomes particularly clear with trigger factors such as disturbed energy-supply or energy-utilization, including O_2_ withdrawal, combined O_2_ glucose withdrawal, pharmacological inhibition of Na^+^/K^+^ ATPase, or mitochondrial poisoning/uncoupling (Andrew et al., 2022b).

The clinical interest was sparked, when it became clear that SD is not limited to *ex vivo* experimental preparations, but may affect also the (intact) human brain. SD is considered in the context of various neurological disorders and conditions, such as migraine, commotio cerebri, postictal depression, cerebral hypoxia/ischemia, and cerebral hemorrhage (van Harreveld and Stamm, 1954; Lauritzen, 1994; Dreier et al., 2006; Dohmen et al., 2008). Furthermore, it is involved in the development of cortical lesions associated with neurological pathologies (Hartings et al., 2017). For example, a recent case series using continuous invasive neuromonitoring combined with longitudinal neuroimaging has demonstrated the prominent role of SD in humans not only temporally after the manifestation of ischemic brain infarcts, but also before and during their development (Lückl et al., 2018). Unclear remains, however, to what extent reactive oxygen species (ROS) are generated during SD and which particular cellular mechanisms participate.

At the cellular level SD evokes a marked flux of K^+^ into the extracellular space, followed by a massive influx of Na^+^, Ca^2+^ and Cl^−^, causing near complete depolarization and swelling of neurons and glial cells (Kraig and Nicholson, 1978; Hansen and Olsen, 1980; Hansen and Zeuthen, 1981). In this condition, which has been termed cytotoxic edema, the electrical resistance of the tissue increases and profound ultrastructural changes occur especially in the dendrites (van Harreveld and Ochs, 1957; Dreier et al., 2018; Kirov et al., 2020). Multiple brief SDs are tolerated by brain tissue without challenging neuronal viability (Nedergaard and Hansen, 1988), but prolonged SD eventually causes neuronal death (Kawasaki et al., 1988). In this process, cellular Ca^2+^-load and metabolic stress seem crucial (Jing et al., 1991; Hartings et al., 2017; Andrew et al., 2022a). However, redox alterations and ROS formation may also need to be considered (Andrew et al., 2022a).

By distorting ionic homeostasis, SD evokes a serious metabolic burden, as indicated by markedly elevated glucose utilization (Shinohara et al., 1979), mitochondrial depolarization (Bahar et al., 2000) and a depletion of ATP on the tissue (Mies and Paschen, 1984) and cellular/neuronal level (unrefereed preprint: Schoknecht, K., Baeza-Lehnert, F., Hirrlinger, J., Dreier, J. P., Eilers J. (2024). Spreading depolarizations exhaust neuronal ATP in a model of cerebral ischemia. bioRxiv, 2024: 605834. doi: https://doi.org/10.1101/2024.07.30.605834). This metabolic burden may then contribute to intensified ROS formation. Indeed, antioxidants (AO) may protect against SD induction, suggesting that ROS may modulate SD induction and/or propagation (Grinberg et al., 2012; Mendes-da-Silva et al., 2014). Direct proof of ROS formation and oxidative stress during SD was obtained in mouse hippocampal slice-cultures by CellROX imaging upon electrically-evoked SD (Grinberg et al., 2012) and in rat neocortex by measuring lipid peroxidation upon K^+^-induced SD (Shatillo et al., 2013), recording MitoSOX fluorescence during brain- trauma-induced SDs (Aboghazleh et al., 2021), or quantifying superoxide dismutase activity together with HPLC analysis of H_2_O_2_ contents in microdialysates upon K^+^-induced SD (Viggiano et al., 2011). For technical reasons, these analyses assessed, however, only a single time point after the tissue had undergone SD. Therefore, the detailed spatiotemporal dynamics of ROS formation during SD remained unclear. A first time-resolved assessment of ROS formation associated with cortical spreading ischemia and cortical spreading depression was based on lucigenin chemiluminescence in the cortex of anesthetized rats, confirming superoxide formation as a consequence of spreading ischemia but not K^+^-induced spreading depression (Dreier et al., 1998).

An intact redox balance is essential for the maintenance of cellular homeostasis. This fragile equilibrium may be distorted, however, by excessive ROS generation, thereby modulating cellular responsiveness, challenging proper cell function or even cellular viability. Based on reactivity profiling, 890 human proteins may be functionally controlled by thiol-based redox modulation (Weerapana et al., 2010). As we demonstrated earlier, also the susceptibility to SD is modulated by redox alterations, with sulfhydryl-reducing conditions favoring and sulfhydryl-oxidizing conditions postponing the onset of hypoxia-induced SD (HSD) (Hepp et al., 2005; Hepp and Müller, 2008). It therefore seems worthwhile taking a closer look at ROS generation and neuronal redox alterations during SD.

The reliable monitoring of subcellular redox conditions in living tissue demands state-of-the-art optical redox sensors, such as the genetically-encoded reduction/oxidation sensitive green fluorescent proteins (roGFPs), which respond reversibly to oxidation as well as reduction and can be expressed in different cell compartments (Hanson et al., 2004). Furthermore, their excitation-ratiometric properties enable quantitative analyses and a reliable comparison of different preparations and conditions (Meyer and Dick, 2010). Mechanistically, engineered reactive thiols respond to ambient redox conditions. Closing (oxidizing) or opening (reducing) of disulfide bridges induces conformational changes, thereby affecting light absorbance and hence fluorescence emission of roGFP (Hanson et al., 2004). Thereby, dynamic and real-time measurements of cellular redox balance become possible. In detailed tests we confirmed the reliability of roGFP in neuronal preparations (Funke et al., 2011; Can et al., 2017) and finally generated transgenic redox indicator mice stably expressing roGFP1 either within cytosol (roGFPc mice, B6J-Tg(Thy1.2-roGFP1c)2Mmllr) or mitochondrial matrix (roGFPm mice, B6J-Tg(Thy1.2-roGFP1m)1Mmllr) of excitatory projection neurons (Wagener et al., 2016). Using these mice, we here deciphered for a first time SD-related neuronal redox alterations in real time and in a quantitative manner. This clearly proved that different modes of SD are accompanied by a massive neuronal oxidation, which shows a clear Ca^2+^ dependence and involves the generation of ROS by mitochondria as well as non-mitochondrial sources.

## 2 Materials and methods

### 2.1 Preparation

Acute cortico-hippocampal tissue slices were obtained from adult male roGFP mice (Wagener et al., 2016); their average age was 5.8 ± 2.9 months (n = 65). Mice were kept and bred at the central animal facility of the University Medical Center Göttingen. For AO treatment, they received an AO-enriched diet upon weaning. The regular diet (V1124-0, SSNIFF Spezialdiäten, Soest, Germany) was supplemented with 250 mg/kg diet *α*-lipoic acid, 2.5 g/kg diet N-acetylcysteine, and an additional 125 mg/kg diet vitamin E (α-tocopherol).

**Figure 1:**
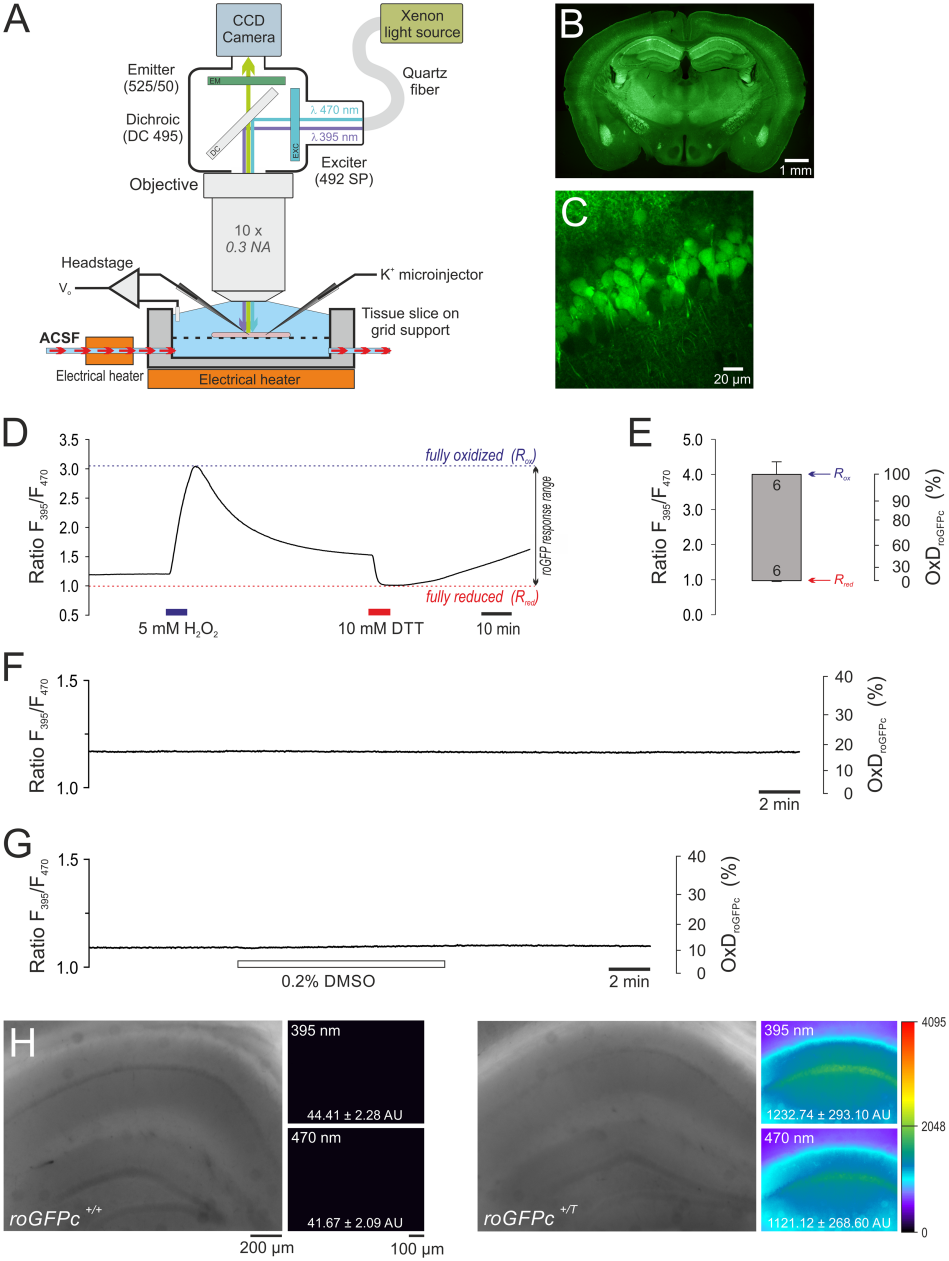
Combined electrophysiological and optical redox imaging experiments in acute slices from roGFPc redox indicator mice. **(A)** Sensitive detection of roGFP fluorescence demands an immersion objective. Thus we were facing the challenge of eliciting SD in submerged brain tissue slices. To ensure tissue viability at the required experimental temperature of 35.5–36.0°C, high flow rates of ACSF (8 mL/min), a slice thickness of 350 μm, and unrestricted access of fresh solution to the upper and lower slice surface turned out to be crucial. SD was triggered by either O_2_ withdrawal, mitochondrial uncoupling or K^+^ microinjection into the slice. **(B)** PFA-fixed transverse slice (30 μm thickness) of a female roGFPc mouse (6 month old) showing the widespread expression of the redox indicator. Note the particularly high expression in hippocampus and the CA1 subfield. The displayed overview is a stitched composition of 12 smaller images; roGFPc fluorescence is displayed in pseudo-colors. **(C)** Close-up view of the CA1 pyramidal cell layer confirming the expression of roGFPc in virtually every pyramidal neuron. Image was taken from an acute brain tissue slice (400 μm thickness, 3 weeks old female roGFPc mouse) with a 2-photon imaging system (TrimScope 2, LaVision BioTec) at 740 nm excitation and 195 nm/pixel resolution. **(D)** Response calibration of roGFPc recorded in *stratum pyramidale* of the hippocampal CA1 subfield. It represents the maximum oxidation (R_ox_) and reduction (R_red_) of roGFPc induced by exposure to H_2_O_2_ and DTT, respectively. During calibration, sufficiently long wash-out and recovery times are essential to prevent direct interactions of the two redox-active compounds. **(E)** Determined roGFPc response range. The upper margin of the box represents the averaged R_ox_, the lower margin the averaged R_red_. Error bars represent the respective standard deviations; the number of slices measured is indicated. Based on the determined calibration parameters, the average degree of roGFPc oxidation (OxD_roGFPc_) is calculated for quantitative redox analysis. **(F)** Control recording confirming that repeated illumination *per se* does not evoke any obvious changes in roGFPc ratio. In the displayed experiment a 3-fold increased frame rate of 0.33 Hz was applied. **(G)** The solvent DMSO (0.2%, 15 min) did not markedly affect roGFPc fluorescence. **(H)** Comparing slices from roGFPc transgenic (*roGFPc^+/T^*) and non-transgenic (*roGFPc^+/+^*) male mice (5 months old siblings) confirms that a contribution of tissue autofluorescence is of no concern. Hardly any fluorescence could be detected in non-transgenic slices. Overview images were taken with an 5x objective and white light illumination. Fluorescence images were acquired under imaging conditions using the 10x water immersion objective. Excitation wavelengths are indicated, and the averaged pixel intensities of the entire fluorescence image are reported in arbitrary units (AU). Fluorescence intensities are displayed in a 12 bit pseudocolor palette spanning 4,096 intensity levels.

Mice were decapitated under deep anesthesia (ether or isoflurane), the brain was isolated and submerged in ice-cold artificial cerebrospinal fluid (ACSF) for 2–3 min. Next, 350 μm-thick coronal tissue slices were cut (752 M Vibroslice, Campden Instruments) and divided in the sagittal midline. These hemi-slices were submerged in a storage chamber with oxygenated regular ACSF (20–22°C) for ≥90 min before the respective recordings were started. Combined electrophysiological and fluorescence recordings were conducted in a submersion-style chamber, which was custom-made by our mechanical workshop to meet the specific technical requirements of our experiments (Figure 1A). The chamber was heated to 35.5–36.0°C and constantly supplied with fresh oxygenated ACSF.

### 2.2 Solutions

Unless stated differently, chemicals were obtained from Sigma-Aldrich. Regular ACSF contained (in mM): 130 NaCl, 3.5 KCl, 1.25 NaH_2_PO_4_, 24 NaHCO_3_, 1.2 CaCl_2_, 1.2 MgSO_4_, and 10 dextrose. The conditioning ACSF used to facilitate SD induction contained a moderately increased K^+^ concentration (8 mM) and was slightly hypo-osmolar (only 110 mM NaCl) (Huang et al., 1997; Funke et al., 2009). In nominally-free Ca^2+^ solutions, CaCl_2_ was omitted from the ACSF. All solutions were constantly aerated with carbogen (95% O_2_, 5% CO_2_) to maintain pH 7.4. FCCP (carbonyl cyanide 4-(trifluoromethoxy) phenylhydrazone, Tocris), and DPI (diphenyleneiodonium, Tocris) were dissolved in DMSO (dimethyl sulfoxide) as 10 mM stocks and kept frozen (−20°C). Final DMSO contents during the experiments were ≤ 0.2%. Allopurinol was directly dissolved in conditioning ACSF shorty before use.

### 2.3 Optical recordings

Quantitative redox imaging was performed by excitation-ratiometric imaging of roGFP fluorescence (Funke et al., 2011; Wagener et al., 2016). The imaging system was composed of an upright microscope (Axiotech, Zeiss), a switchable xenon light source (TILL Photonics Polychrome V, ThermoFisher Scientific) and a sensitive CCD camera (Imago QE, PCO Imaging). Slices were viewed with a 5×, 0.13NA objective (Epiplan, Zeiss) for proper positioning in the chamber and electrode insertion. For fluorescence imaging, a low magnification 10×, 0.3NA water immersion objective was chosen (Achroplan, Zeiss).

Taking advantage of the rapid wavelength-switching capabilities of the computer-controlled imaging system, roGFP was alternately excited at 395 and 475 nm and the respective fluorescence recorded at the tissue level at low magnification (TILLvision 4.5 software; ThermoFisher Scientific). Exposure times were 12 ms for each wavelength, 2×2 pixel binning was applied, and ratiometric image pairs were recorded every 10 s (0.1 Hz frame rate). The fluorescence ratio F_395_/F_470_ was calculated in real-time for pre-defined regions of interest (ROIs, ~20×40 μm) near the recording electrode and displayed as line plots. An increased fluorescence ratio F_395_/F_470_ indicates oxidation of roGFP, a decreased ratio indicates reduction of roGFP. Post-experimental image analysis was performed with TILLvision 4.5 and Metamorph offline 7.8 (Molecular Devices).

For quantitative recordings we calibrated the ratiometric roGFPc responses in the CA1 pyramidal cell layer (Figures 1D,E). The ratio representing full oxidation (R_ox_) was determined by exposing the slice to 5 mM H_2_O_2_ (7 min), the ratio representing full roGFP reduction (R_red_) was determined by treatment with 10 mM DTT (7 min). Monitoring both responses in the same slice, provides also the ratio of fluorescence intensities (F470_ox_/F470_red_). Once calibrated, the relative degrees of roGFP oxidation (OxD_roGFP_) were then calculated for the roGFP1 sensor under our recording conditions (Meyer and Dick, 2010; Wagener et al., 2016):


$${OxD}_{\mathrm{roGFP}}=\frac{R-R_{\mathrm{red}}}{\frac{F470_{\mathrm{ox}}}{F470_{\mathrm{red}}}\;\left(R_{\mathrm{ox}}-R\right)+\left(R-R_{\mathrm{red}}\right)}$$

### 2.4 Electrophysiological recordings

Extracellular DC potentials were recorded with a custom-built amplifier and glass microelectrodes inserted in CA1 *stratum radiatum*. Thin-walled borosilicate glass capillaries (GC150TF-10, Harvard Apparatus) were pulled on a P-97 electrode puller (Sutter Instruments), filled with ACSF, and their tips trimmed to a resistance of ~5 MΩ. DC potentials were sampled/digitized at 100 Hz (Axon-Instruments Digitizer 1322A, PClamp 9.0 software, Molecular Devices) and analyzed offline. In parallel, the TTL-pulses triggering image acquisition by the CCD camera were recorded, to synchronize the optical and electrophysiological signatures of SD during data analysis. In some slices, field excitatory postsynaptic potentials (fEPSPs) were evoked orthodromically every 30 s by 1.0–1.5 mA unipolar stimuli of 0.1 ms duration (S88 Grass Stimulator with PSIU6 stimulus isolation units, 50 μm steel wire electrode). In these recordings, a sampling rate of 20 kHz was used. For reliable stimulation of the submerged tissue slices, the steel microwire electrodes – except for their very tips – were insulated with 3 layers of polyurethane paint (urethan 71, CRC Industries). All DC potential amplitudes were measured between the respective pre-treatment baseline and the nadir of SD. As SD onset we defined the sudden negative DC potential deflection (ΔV_o_); only rapid deflections were considered as full-blown SDs.

Normoxic SD was induced by local injection of 3 M KCl microdroplets (PDES-02DX microinjector, NPI Electronic, Tamm, Germany; 1.5 s pressure pulse of 0.25 bar). HSD was triggered by 95% N_2_, 5% CO_2_ aerated conditioning ACSF including also 2 mM sodium sulfite (Na_2_SO_3_) to remove any residual O_2_ and prevent a re-oxygenation within the tubing system and the recording chamber (Flyvbjerg et al., 1993; Funke et al., 2011). Chemically-induced SDs were evoked by the mitochondrial uncoupler FCCP (2 μM) (Gerich et al., 2006).

### 2.5 Statistics

Reported data were collected from 65 mice, typically using 3–4 slices from each brain. To ensure the independence of observations, each experimental paradigm was conducted on at least 3 different mice. For a first screening of data, an outlier test (ROUT, Q = 1%, GraphPad Prism 9.5.1.) was performed and only those slices were included into analysis, which presented reasonable optical and electrophysiological data in the combined recordings.

Results are reported as mean ± standard deviation, n represents the number of slices analyzed. The scatter of the individual data points is displayed in the diagrams. All treatment-related changes are referred to the respective control conditions. Normal distribution was verified by the Kolmogorov–Smirnov test. The statistical significance of optical/electrophysiological data sets was assessed in one-sample Student’s t-tests at a significance level of 5%, by comparing any evoked differences against pre-treatment control conditions (defined as unity). For the comparison of several groups/treatment conditions, one way ANOVA (analysis of variance) followed by Holm-Šídák comparisons were used. In the case of non-normally distributed data sets, a Kruskal-Wallis One Way Analysis of Variance on Ranks, followed by Dunn’s test comparisons were applied. In the diagrams significant changes are indicated by asterisks (* *p* < 0.05; ** *p* < 0.01, *** *p* < 0.001) and the statistical test applied is stated. For data processing and statistical calculations Excel (Office 2016), Sigma Stat 3.5 (Systat Software), and GraphPad Prism 9.5.1 (GraphPad, Inc.) were used.

## 3 Results

### 3.1 Optimizing recording conditions and slice chamber design

Establishing quantitative redox imaging during SD, we were facing the two challenges of efficient fluorescence detection and the high temperature required to induce SD. To guarantee highest possible fluorescence detection sensitivity, the slices had to be submerged. Yet, this condition complicates the maintenance of brain tissue viability and the reliable induction of SD. Therefore, the very first task was the design of an experimental chamber for the electro-optical recordings at the required temperature of 35.5–36.0°C (Figure 1A).

Based on earlier experience and advice of colleagues who regularly study SD under submerged conditions (Reinhart and Shuttleworth, 2018), acute brain tissue slices were prepared at a thickness of 350 μm and positioned on a supportive nylon mesh to assure undisturbed contact of both slice surfaces to fresh ACSF. Also high flow rates of 8 mL ACSF/min were applied to guarantee sufficient O_2_ supply of the submerged slices (Hájos et al., 2009; Reinhart and Shuttleworth, 2018).

### 3.2 Validation and calibration of roGFP responses

Quantitative redox imaging demands a proper validation and calibration of the redox sensor in the respective tissue. Based on the expression of roGFPc in the cytosol of excitatory neurons, we chose CA1 *stratum pyramidale* for all experiments, as it showed the highest roGFP expression (Figures 1B,C). Due to the massive cell swelling during SD, redox imaging was not performed at the single-cell level, but at low magnification in defined tissue volumes, i.e., ROIs of about 20×40 μm size, containing several roGFP-expressing neurons.

At first we calibrated the ratiometric response range (Figures 1D,E) and determined the maximum oxidation (R_ox_) and maximum reduction (R_red_) of roGFPc by exposing the slices to 5 mM H_2_O_2_ and 10 mM DTT (7 min each). In detail, an R_ox_ of 4.000 ± 0.370, an R_red_ of 0.972 ± 0.013, and an instrument factor F470_ox_/F470_red_ of 0.332 ± 0.035 (*n* = 6 each) were obtained (Figures 1D,E). Under resting control conditions, the roGFPc ratio within CA1 *stratum pyramidale* averaged 1.174 ± 0.080 (*n* = 98). Based on the calibrations, this corresponds to an average degree of roGFPc oxidation (OxD_roGFPc_) of 17.4 ± 5.8% (*n* = 98).

Next, we validated the stability and reliability of roGFP under the chosen experimental conditions. Brain tissue slices were exposed to the imaging protocol without any other treatments, and in part the imaging frame rate was even increased from 0.1 Hz to 0.33 Hz. As expected, up to 1,000 cycles of ratiometric excitation did not evoke marked OxD_roGFPc_ alterations in the hippocampal CA1 subfield (2.43 ± 2.44 percentage points as compared to control conditions, *n* = 4, range − 0.32 – 5.04 percentage points). This confirms that the frequent illumination and the associated recording conditions do not interfere with roGFP integrity and/or the redox conditions in the studied tissue volumes (Figure 1F).

Furthermore, we addressed potential side-effects of the drug solvent DMSO. Any marked effects of DMSO (0.2%, 15 min) on the redox balance could not be observed within the CA1 pyramidal cell layer (*n* = 3, Figure 1G). Compared to baseline control conditions, OxD_roGFPc_ only slightly increased during the 15 min of DMSO administration by 1.16 ± 0.86 percentage points.

Finally, we ruled out potential contributions of tissue autofluorescence, by imaging wildtype slices devoid of roGFPc (*n* = 3). With identical 395 nm and 470 nm imaging settings applied, hardly any fluorescence was detectable in non-transgenic slices, confirming that roGFP fluorescence is at least 25-fold more intense than any background autofluorescence of the hippocampal tissue (Figure 1H).

### 3.3 Spreading depolarization is paralleled by an oxidizing redox shift

To characterize the redox changes associated with SD, we induced SDs by either an increase in the extracellular K^+^ concentration, O_2_ withdrawal or mitochondrial uncoupling, thereby covering a spectrum of normoxic, hypoxia-and chemically-induced SDs. For reliable SD induction, conditioning ACSF was used in all recordings (see solutions).

**Figure 2:**
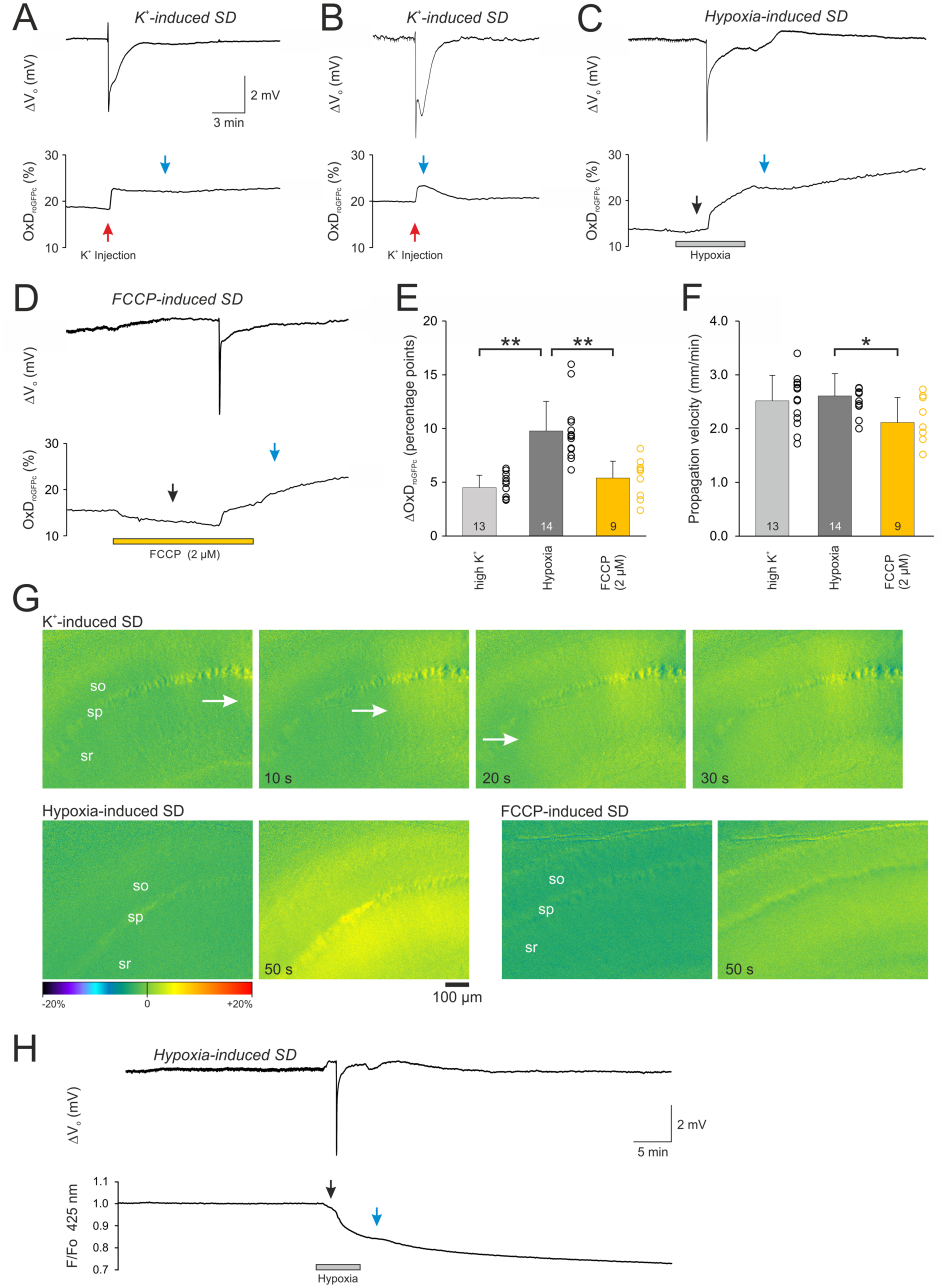
The different modes of SD evoke a pronounced oxidative shift in cellular redox balance. **(A,B)** Normoxic, K^+^-induced SD recorded as extracellular DC potential deflection (ΔV_o_) with the associated redox alterations expressed as degree of roGFPc oxidation (OxD_roGFPc_). Note the variable reversibility of the redox changes. Red arrows indicate the time point of K^+^ microinjection, blue arrows the time point at which roGFPc oxidation was quantified. **(C)** Hypoxia (N_2_ + 2 mM sulfite)-induced SD with the associated irreversible redox alterations. Note that a moderate roGFPc oxidation started already before SD onset (black arrow). **(D)** FCCP-induced SD with associated irreversible redox alterations. Administration of FCCP and the resulting mitochondrial uncoupling evoked a noticeable reducing shift before SD occurred (black arrow). **(E)** Magnitude of the SD-related oxidative changes in the neuronal cytosolic redox balance. Plotted are the changes in the degree of roGFP oxidation (ΔOxD_roGFPc_) as mean ± standard deviation; the number of slices analyzed is reported for each bar. The dots next to each bar represent the scatter of the underlying data points. Note that most pronounced redox changes occurred during HSD. The extent of roGFPc oxidation was quantified either at the peak or in those conditions not reaching a clear peak, 300 s after SD onset, see blue arrow marks. **(F)** Propagation velocity of the oxidative wavefront during the different modes of SD. Propagation of SD was lowest in the case of FCCP-induced SDs. Asterisks indicate significantly different changes (* *p* < 0.05, ** *p* < 0.01; one way ANOVA and Holm-Šídák comparison versus hypoxic SD). **(G)** Image series depicting the onset and propagation of the SD-associated redox wavefronts for the conditions of K^+^-, hypoxia-, and FCCP-induced SDs. The hippocampal layers are indicated (so *stratum oriens*, sp *stratum pyramidale*, sr *stratum radiatum*). Time tags of the respective images indicate the time passed from the first images displayed. Scale bar is identical for all images. The white arrows indicate the propagating wavefront. Images are subtraction images (first image of the time series subtracted from all subsequent images). The displayed changes in roGFPc fluorescence ratios (± 20% range) are displayed in pseudo-colors, with warmer colors indicating a change toward oxidation (i.e., increased roGFPs ratios). **(H)** Imaging roGFPc fluorescence at its isosbestic point (425 nm excitation) reveals the non-redox-related optical changes during HSD, most of which can be expected to be cell swelling diluting the cytosolic fluorophore concentration. A recovery of the fluorescence decrease did not occur upon reoxygenation.

Normoxic, K^+^-induced SDs were triggered by local K^+^ micro injection into the slice (*stratum radiatum*) ~100 μm next to the electrical recording site. After a short delay of a few seconds (range 3–7 s) SD occurred, showing the characteristic negative extracellular DC potential shift (ΔV_o_), which averaged −4.6 ± 2.4 mV (*n* = 13; see Table 1). It was accompanied by a more slowly developing increase in roGFPc ratio, i.e., an oxidizing shift in cytosolic redox balance, which brought OxD roGFP to 21.8 ± 8.1% (*n* = 13; Figures 2A,B,E). Upon recovery of the ΔV_o_, the roGFP oxidation remained at its oxidized level in 8 out of 13 slices studied, hardly showing any recovery during the remaining ~30 min of recording (Figure 2A). In the other 5 slices, a partial or even near-complete recovery of the SD-related redox changes was observed (Figure 2B). Inspecting the recorded image series revealed that the oxidative wavefront propagated at a velocity of 2.52 ± 0.47 mm/min (*n* = 13; Figures 2F,G).

**Table 1:** Electrical parameters determined for the different types of SD, i.e., amplitude (ΔV_o_), time to onset (Δt), and duration (t1/2) of the extracellular DC potential deflection.

Mode of SD	ΔV_o_ [mV]	Δt [min]	t ½ [s]	Number of slices
K^+^-induced (normoxic)	−4.60 ± 2.35	---	91.52 ± 72.38 *	13
Hypoxia-induced (N_2_ + 2 mM sulfite)	−3.54 ± 1.21	3.96 ± 0.97	13.92 ± 8.26	14
FCCP-induced (mitochondrial uncoupling)	−4.55 ± 1.76	7.01 ± 2.65 *	8.40 ± 4.54	9

In the case of K^+^-induced SD, an accurate time to onset could not be determined; the normoxic SDs typically occurred 3–7 s upon K^+^ microinjection. Listed data are mean ± standard deviations. Asterisks identify statistically significant differences as compared to HSD (*p < 0.05; Kruskal-Wallis one way ANOVA on ranks, followed by Dunn’s Test multiple comparison). Accordingly, K^+^-induced SDs lasted longer than HSDs, whereas FCCP-induced SDs occurred markedly later than HSDs.

In the case of HSD, a moderate oxidation started during application of the hypoxic solution already before SD onset, shifting OxD_roGFPc_ from its prehypoxic baseline of 16.4 ± 4.1% to 17.6 ± 3.9% (*n* = 14, Figure 2C). SD occurred within 3.96 ± 0.97 min of hypoxia and its amplitude averaged −3.5 ± 1.2 mV (*n* = 14, Table 1). It was paralleled by a marked and more rapid further oxidation to an OxD_roGFPc_ of 26.2 ± 3.6% (*n* = 14). Whereas the oxidation was more intense than during normoxic SD (Figure 2E), the oxidative wavefront propagation velocity was almost identical (2.61 ± 0.41 mm/min, *n* = 14; Figures 2F,G). A clear plateau was, however, not reached in the case of HSD; even though wash-out of the hypoxic solution was started 3 min after SD onset, roGFP typically continued to oxidize further for the remainder of the experiment. This continued oxidation obviously indicates a loss of viability, as the O_2_ scavenger Na_2_SO_3_ included in the hypoxia-inducing solution could not be removed from the slices sufficiently fast, hampering a successful re-oxygenation of the vulnerable tissue.

The mitochondrial uncoupler FCCP (2 μM) caused an initial decrease (reduction) of the roGFP1 ratio, shifting OxD_roGFPc_ from its pre-treatment baseline of 15.6 ± 5.3% to 13.1 ± 5.0% (*n* = 9). This indicates that basal mitochondrial ROS production constantly challenges cytosolic redox balance (Figure 2D). Within 7.0 ± 2.7 min of FCCP treatment the chemically-induced SD occurred, and the ΔV_o_ amplitude averaged −4.6 ± 1.8 mV (*n* = 9). In parallel a clear roGFP oxidation occurred, shifting OxD_roGFPc_ to 21.0 ± 5.6% (Figure 2D). The magnitude of roGFPc oxidation was similar to normoxic SD, but less intense than during HSD (Figure 2E). The propagation velocity of the oxidative wavefront averaged 2.11 ± 0.47 mm/min (*n* = 9; Figures 2F,G), being somewhat slower than during hypoxia-and K^+^- induced SDs. Wash-out of FCCP was started 3 min after SD onset, but the SD-related oxidation did not recover. Instead, roGFPc was oxidized further for the rest of the experiment (Figure 2D), similar to the conditions during HSD.

As SD is accompanied by marked changes in the optical properties of the tissue (intrinsic optical signal, IOS), we also performed experiments in which roGFPc was excited at its isosbestic point of 425 nm (Figure 2H). Induction of hypoxia resulted in a moderate decline of the recorded roGFP fluorescence already before HSD onset, averaging 3.8 ± 0.9% (*n* = 4) as compared to prehypoxic baseline conditions. Occurrence of HSD was accompanied by a further fluorescence decline, which at the 300 s second time point used for analysis averaged 17.7 ± 2.8% (*n* = 4). When oxygenation was restored, a recovery was not observed and 40 min upon reoxygenation the fluorescence decline measured 30.0 ± 2.6% (*n* = 4). As roGFPc was, however, excited ratiometrically for redox analyses, these intrinsic changes in fluorescence intensity – which can be assumed to reflect mostly cell swelling and the associated dilution of cytosolic roGFP content – are minimized by the ratiometric approach.

### 3.4 Recovery from SD

**Figure 3:**
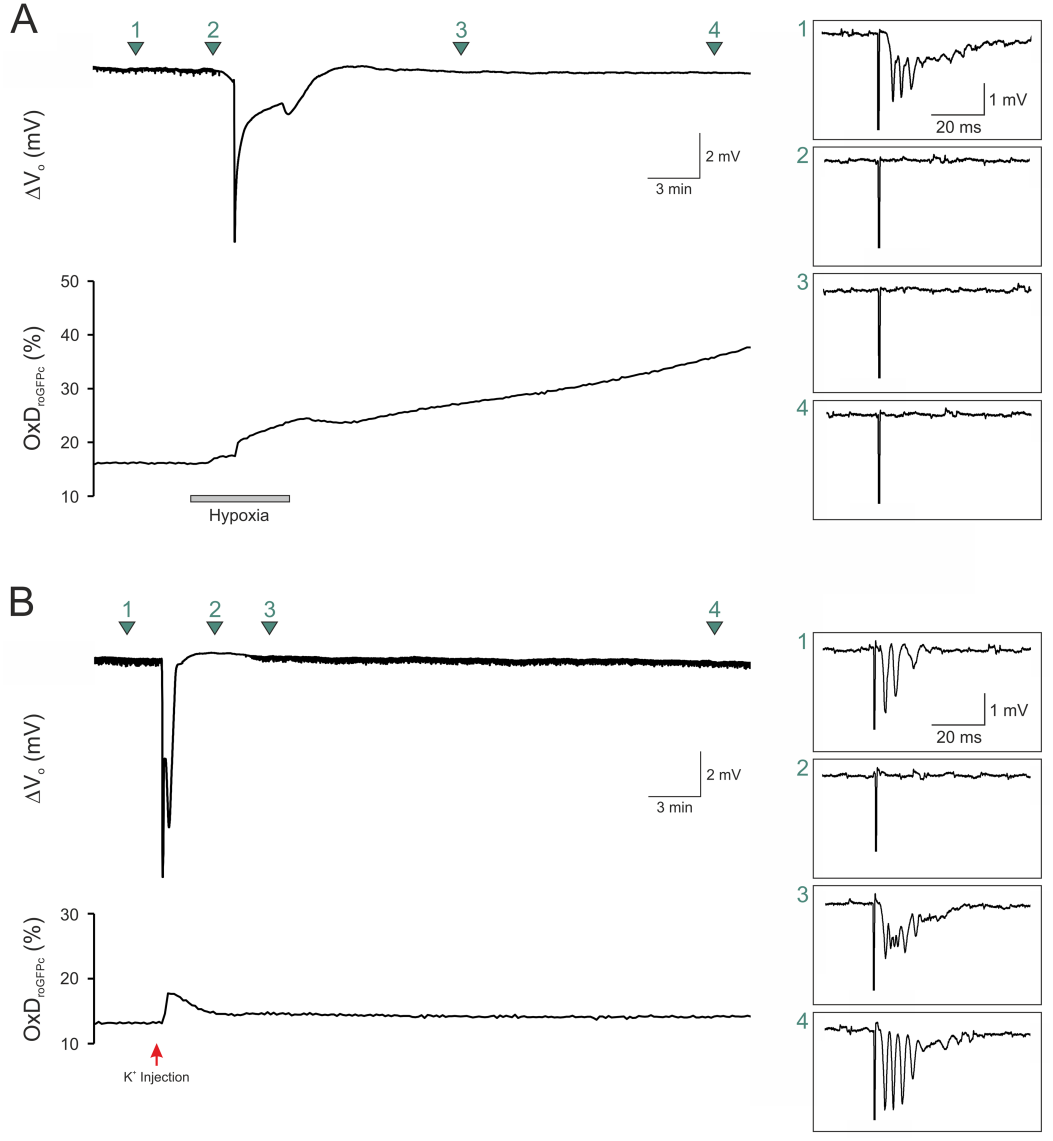
Sustained roGFP oxidation is accompanied by a loss of cellular viability. **(A)** Eliciting fEPSPs (1.0 mA orthodromic stimuli) revealed a loss of synaptic function during hypoxia even before HSD onset. Upon reoxygenation, neither the roGFPc oxidation nor synapses recovered. Green arrow marks indicate the exact time points at which the displayed fEPSPs were recorded. Stimulation artifacts were truncated to fit the boxes. **(B)** Normoxic, K^+^-induced SD also blocked synaptic function, but within minutes, roGFPc oxidation as well as fEPSPs (0.5 mA orthodromic stimuli) fully recovered. Red arrow indicates the time point of K^+^ microinjection.

The irreversible roGFPc oxidation observed during the conditions of hypoxia-and FCCP-induced SDs suggests an impairment of cellular viability. To confirm this assumption, we evoked field excitatory postsynaptic potentials (fEPSPs) by Schaffer-collateral stimulation; these orthodromic stimuli were applied every 30 s for the entire experiment. As expected, O_2_ withdrawal abolished the evoked responses already before HSD onset. Upon reoxygenation, fEPSPs failed to recover whereas roGFPc continued to become more oxidized (*n* = 4; Figure 3A). Eliciting fEPSPs during K^+^-induced SD showed that fEPSPs vanished during the course of SD but then started to recover within a few minutes (*n* = 3), finally reaching their original amplitude while also the roGFPc oxidation recovered (Figure 3B).

### 3.5 Mechanistic sources of the SD-associated cytosolic oxidation

Various cellular sources generate ROS, thereby challenging cellular redox balance. These include besides mitochondria especially NADPH oxidase and xanthine oxidase (Dröge, 2002; Finkel, 2011). Also, pronounced cellular Ca^2+^ load may drive cell-endogenous ROS production (Görlach et al., 2015). The following experiments therefore aimed to clarify which of these sources contribute to the SD-related oxidation.

**Figure 4:**
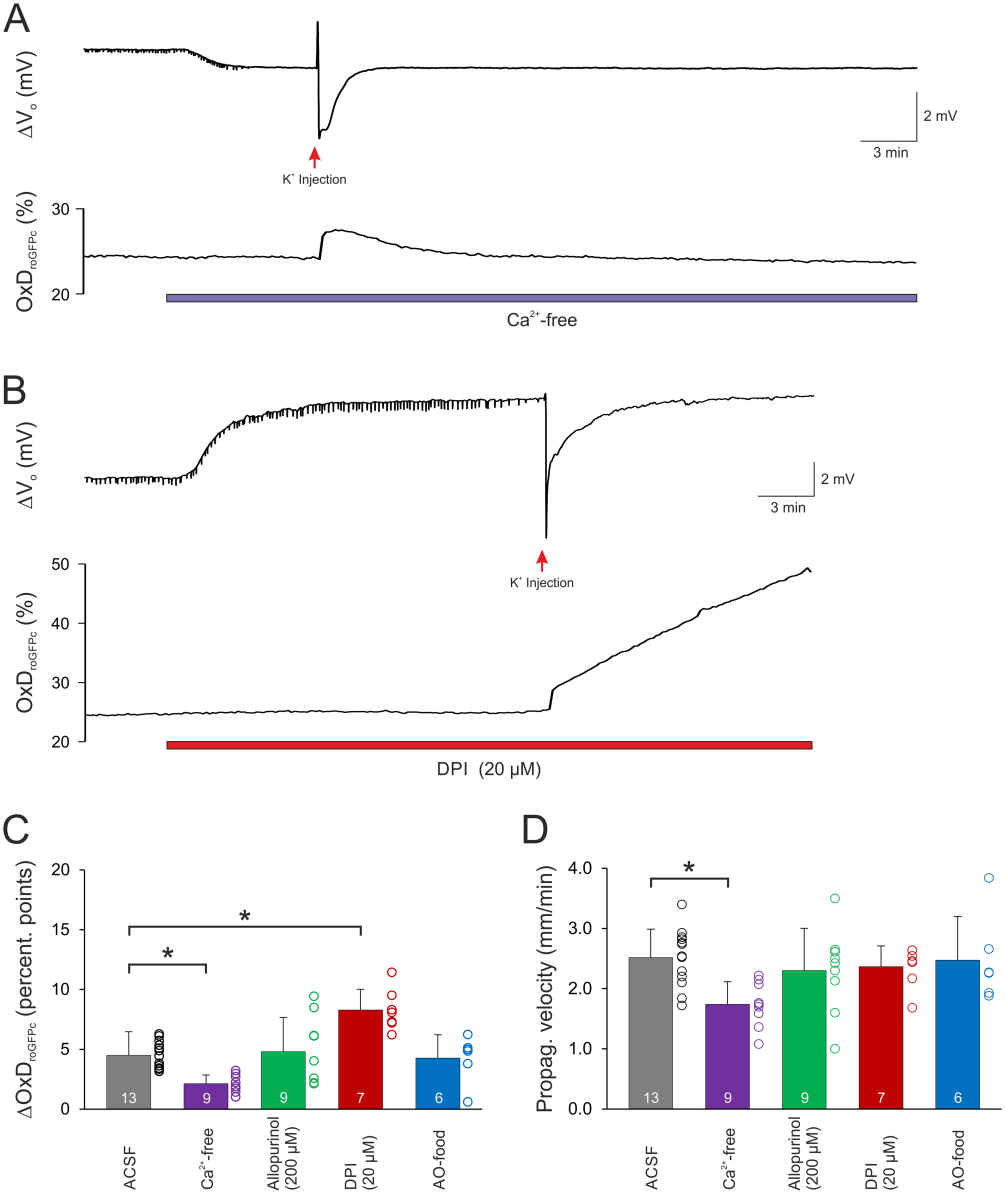
Modulation of normoxic SD-related redox changes. **(A)** Ca^2+^ withdrawal dampened the roGFPc oxidation associated with normoxic SD. Furthermore, the oxidative shift became fully reversible. **(B)** Upon pre-treatment with DPI (20 μM, 15 min), the SD-associated roGFPc oxidation became irreversible. Wash-in of DPI caused a positive shift of the extracellular DC potential, which is due to an offset induced by this compound at the reference electrode; it occurred in all experiments with DPI and averaged 5.3 ± 3.7 mV (*n* = 17). **(C)** Magnitude of the normoxic SD-associated oxidative shift, quantified as changes in OxD_roGFPc_. In Ca^2+^-free solutions, the oxidative shift was significantly less pronounced, in the presence of DPI it became more intense (*p < 0.05; one way ANOVA on ranks with Dunn’s test multiple comparisons versus ACSF). **(D)** The propagation velocity of the oxidative wavefront during normoxic SD was not significantly affected by most treatments. Only upon withdrawal of extracellular Ca^2+^ a slower propagation was observed (*p < 0.05; one way ANOVA and Holm-Šídák multiple comparisons versus ACSF).

**Figure 5:**
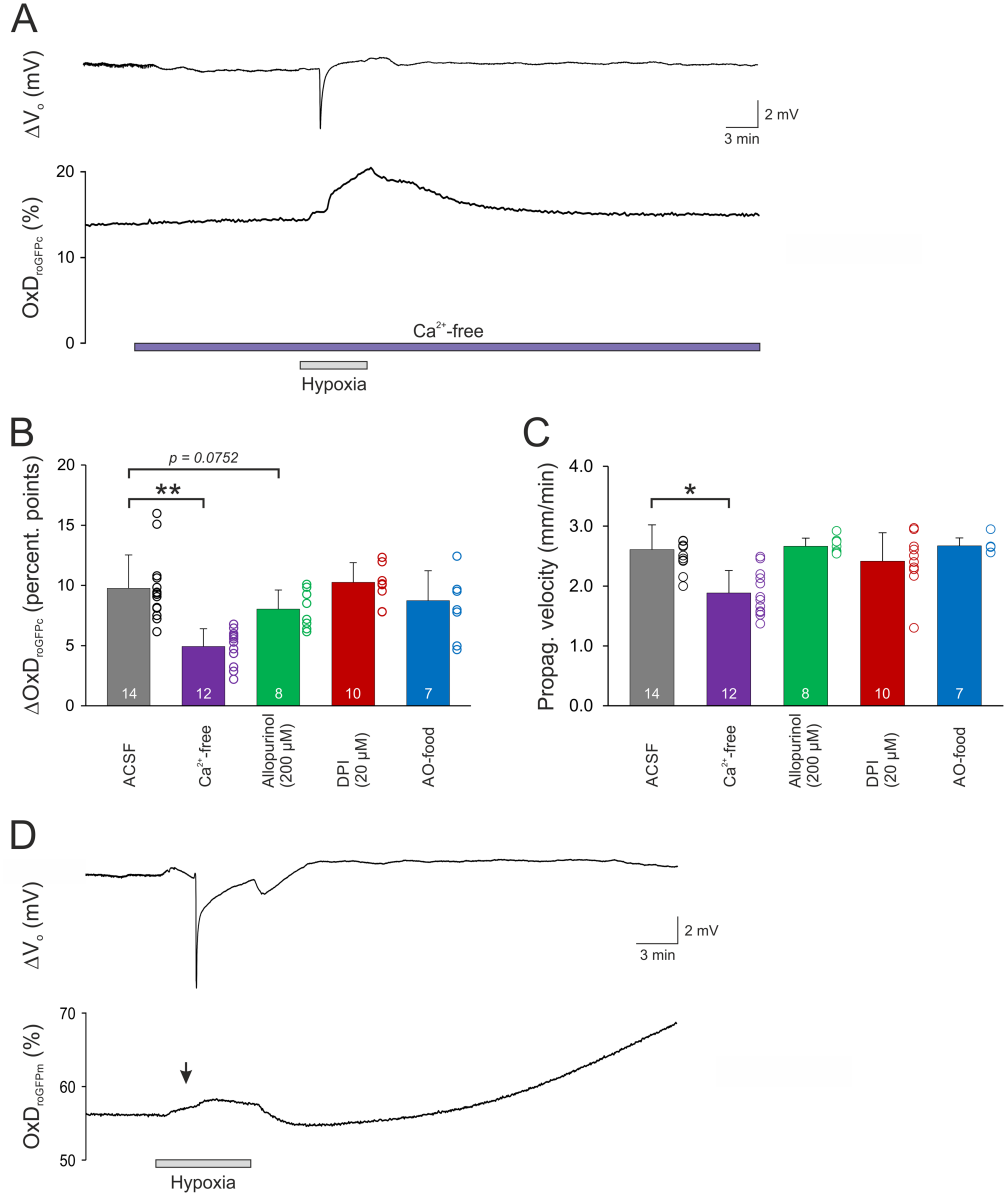
Modulation of HSD-related redox changes. **(A)** In the absence of extracellular Ca^2+^ the magnitude of the roGFPc oxidation associated with HSD was significantly dampened and became largely reversible upon reoxygenation. **(B)** Statistical summary of the redox alterations confirms a significant decrease in Ca^2+^ free solution and a tendency of dampening upon allopurinol treatment. The number of slices studied is indicated for each group (**p < 0.01; one way ANOVA with Holm-Šídák multiple comparisons versus ACSF). **(C)** The propagation velocity of the oxidative wavefront was also decreased in Ca^2+^ free solutions, whereas the other treatments did not mediate any significant changes. The number of slices is reported (*p < 0.05, Kruskal Wallis one way ANOVA on ranks with Dunn’s test multiple comparisons versus ACSF). **(D)** HSD-induced oxidation of mitochondrial matrix. Mice expressing roGFP in mitochondria (roGFPm mice) revealed that mitochondrial matrix shows markedly less intense redox changes than cytosol. Induction of hypoxia evoked already a moderate oxidation (see black arrow mark). As HSD occurred, this initial oxidation became only slightly more intense and it quickly recovered upon reoxygenation, before mitochondrial matrix then underwent a secondary, slowly progressing oxidation for the remaining duration of the experiment. Note that redox baseline-conditions in mitochondrial matrix are more oxidized than in cytosol.

To assess the involvement of cellular Ca^2+^ influx via voltage-and transmitter-activated Ca^2+^ channels, slices were pretreated for 15 min with Ca^2+^-free conditioning ACSF. It should be noted here, that this treatment does not prevent Ca^2+^ release from intracellular stores, which however only moderately contributes to the massive SD-related cellular Ca^2+^ load. Ca^2+^ withdrawal *per se* did not affect OxD_roGFPc_ baseline conditions (*n* = 21; see Table 2). However, when K^+^-induced SDs were induced in the absence of extracellular Ca^2+^, roGFP oxidation was significantly dampened. The OxD_roGFPc_ rose by only 2.13 ± 0.73 percentage points (*n* = 9) and more importantly it became fully reversible (Figures 4A,C). Furthermore, the propagation velocity of the oxidative wavefront dropped to 1.74 ± 0.37 mm/min (*n* = 9; Figure 4D). The oxidative shift during HSD was also significantly decreased in Ca^2+^-free solutions. The OxD_roGFPc_ increase measured only 4.92 ± 1.47 percentage points (*n* = 12) and fully recovered upon reoxygenation (Figures 5A,B). In addition, the propagation velocity decreased to 1.88 ± 0.38 mm/min (*n* = 12; Figure 5C). The electrophysiological parameters of the DC potential shifts during KSD and HSD were not significantly affected in the absence of extracellular Ca^2+^ (see Table 3).

**Table 2:** Redox alterations during drug application.

Drug-induced redox changes (ΔOxD_roGFPc_) in percentage points				
Treatment	Mean	Stand. Dev.	*n*	Significance
Imaging only (1,000 frames)	2.43	2.44	4	No
DMSO (0.2%), 15 min	1.16	0.86	3	*
FCCP (2 μM)	−2.480	1.241	9	***
Ca^2+^ withdrawal, 15 min	0.213	0.908	21	No
Allopurinol (200 μM), 15 min	−0.338	1.140	17	No
DPI (20 μM), 15 min	0.209	1.374	17	No
95% N_2_, 5% CO_2_ + 2 mM sulfite	1.217	1.228	14	**

Listed are the averaged changes in OxD_roGFPc_ observed during the respective drug pre-treatments. Statistical significance is indicated (* *p* < 0.05; ** *p* < 0.01, *** *p* < 0.001; one-sample t-test versus unity, i.e., “0”). In the case of hypoxia and FCCP the reported redox changes represent those redox alterations occurring during drug treatment until the time point of SD onset.

**Table 3:** Summary of the characteristic parameters of the negative DC potential deflection (amplitude ΔV_o_; time to onset Δt; duration t ½) that were associated with normoxic and hypoxia-induced SD.

K^+^-induced normoxic SD				
Conditions	ΔV_o_ [mV]	Δt [min]	t ½ [s]	*n*
ACSF	−4.60 ± 2.35	–	91.52 ± 72.38	13
Ca^2+^-free	−3.13 ± 1.51	–	40.14 ± 19.61	9
DPI (20 μM)	−5.02 ± 2.32	–	103.95 ± 74.46	7
Allopurinol (200 μM)	−5.25 ± 3.12	–	49.46 ± 30.89	9
AO-enriched diet	−3.33 ± 2.17	–	58.94 ± 51.85	6

Hypoxia-induced SD				
Conditions	ΔV_o_ [mV]	Δt [min]	t ½ [s]	*n*
ACSF	−3.54 ± 1.21	3.96 ± 0.97	13.92 ± 8.26	14
Ca^2+^-free	−4.99 ± 4.01	3.59 ± 1.03	49.32 ± 45.8	12
DPI (20 μM)	−3.39 ± 1.19	3.75 ± 0.61	6.56 ± 2.30*	10
Allopurinol (200 μM)	−5.67 ± 2.05	4.01 ± 0.60	10.73 ± 6.19	8
AO-enriched diet	−6.05 ± 1.56 *	3.02 ± 0.52	12.94 ± 11.01	7

Normoxic SD was not significantly affected by the different conditions tested, in the case of HSD, DPI treatment significantly shortened the duration of the DC potential deflection, whereas the DC potential amplitude was increased in antioxidant (AO)-fed mice (* *p* < 0.05, Kruskal Wallis one way ANOVA on ranks, Dunn’s test multiple comparisons vs. ACSF; - not determined).

Application of allopurinol, an inhibitor of xanthine oxidase (200 μM, 15 min), did not induce any changes in the resting redox balance of CA1 pyramidal neurons (*n* = 17 slices, Table 2). Induction of normoxic SD in the presence of allopurinol did not modify the ΔV_o_ parameters (Table 3), the oxidizing shift, OxD_roGFPc_ increased by 4.81 ± 2.85 percentage points, or its propagation velocity (*n* = 9; Figures 4C,D).

When HSD was evoked in the presence of allopurinol a tendency toward dampened roGFPc oxidation was observed (*p* = 0.0752, Figure 5B), with OxD_roGFPc_ increasing by 8.04 ± 1.58 percentage points (*n* = 8). The wavefront propagation velocity, however, remained unaffected (Figure 5C). Neither were the electrical HSD parameters affected.

Pretreating slices with the NADPH inhibitor DPI (20 μM, 15 min) did not evoke any significant changes in redox baseline conditions either (*n* = 17 slices). When normoxic K^+^-induced SD was recorded in the presence of DPI, the ΔV_o_ remained unaffected, but the associated redox changes became less reversible, and in part a further slow roGFPc oxidation occurred. Accordingly, the OxD_roGFPc_ changes associated with K^+^-induced SD were intensified, averaging 8.30 ± 1.72% (*n* = 7, Figures 4B,C). The propagation velocity of the oxidative wavefront was not affected (Figure 4D). Inducing HSD in the presence of DPI shortened the duration of the ΔV_o_ (Table 3), but did not reveal any obvious effects on OxD_roGFPc_ or the wavefront propagation (Figures 5B,C).

As a more general approach to improve cellular ROS buffering, some mice received an AO-enriched diet (see Material and Methods). In AO-treated mice, the resting redox baselines in CA1 *stratum pyramidale* corresponded to an OxD_roGFPc_ of 15.6 ± 3.2% (*n* = 13), which tended to be slightly more reduced than in normally fed mice (17.4 ± 5.8%, *n* = 98) but the level of significance was not reached (unpaired two-tailed t-test). Evoking a K^+^-induced SD in these mice did not reveal any significant effects on the electrical or the optical redox parameters of SD (*n* = 6, Figures 4C,D). Likewise, the roGFPc oxidation during HSD or the wavefront propagation were not significantly affected by AO treatment (Figures 5B,C); the amplitude of the DC potential deflection was slightly increased though (*n* = 7; Table 3).

### 3.6 Is SD associated with an oxidation of mitochondria?

As O_2_ withdrawal primarily targets the mitochondria, it remains to be clarified how their redox balance is affected by SD. To solve this question, we used our transgenic roGFPm mice expressing roGFP within mitochondrial matrix (Wagener et al., 2016). For reliable quantitation, we first calibrated roGFPm responses in CA1 *stratum pyramidale* (R_ox_ = 3.601 ± 0.160, R_red_ = 1.021 ± 0.033, F470_ox_/F740_red_ = 0.321 ± 0.017; *n* = 4). Inducing HSD in these mice revealed a clearly distinct redox pattern (Figure 5D). Showing an OxD_roGFPm_ of 67.3 ± 6.7% (*n* = 10), baseline redox conditions in mitochondrial matrix were more oxidized than in cytosol. Upon O_2_ withdrawal, a moderate oxidation of roGFPm was detected before HSD onset. On average, HSD occurred within 2.85 ± 0.73 min of hypoxia and the ΔV_o_ amplitude averaged −4.35 ± 2.20 mV (*n* = 7). During HSD, only a moderate further oxidation of mitochondrial matrix occurred, and OxD_roGFPm_ was shifted to oxidation by only 1.42 ± 1.50 percentage points (*n* = 10; range 0.02–5.33). Upon reoxygenation, matrix redox balance quickly recovered to baseline conditions. At the 300 s analysis time point (blue arrow marks in Figure 2) at which the cytosolic oxidation upon HSD was clearly developed, mitochondrial matrix was already indistinguishable from pre-hypoxic baseline conditions. However, several minutes upon reoxygenation, mitochondrial matrix then underwent a delayed oxidative shift continuing for the remaining duration of the experiment (Figure 5D), similar to what we observed in cytosol during HSD.

## 4 Discussion

Here we present the first assessment of neuronal ROS production and subcellular redox dynamics during K^+^-, hypoxia-and chemically-induced SD. These experiments became possible based on our redox-indicator mice, which stably express roGFP1 in either cytosol or mitochondrial matrix of excitatory projection neurons (Wagener et al., 2016), as well as an optimized recording chamber developed for combined optical and electrical SD recordings in submerged slices.

### 4.1 Advanced properties of roGFP

Among the advantages of roGFP is that these sensors are ratiometric by excitation, reliably quantifying cellular redox conditions largely independent of sensor concentrations, cell volume changes and sensor bleaching (Dooley et al., 2004; Hanson et al., 2004). Being fully embedded into cellular thiol redox balance and responding reversibly to oxidizing and reducing stimuli, roGFP reports cellular thiol redox dynamics in real-time. The equilibration of roGFP with the ambient thiol redox conditions is catalyzed by glutaredoxins and thioredoxins (Dooley et al., 2004; Björnberg et al., 2006). As we confirmed earlier, roGFP1 is hardly affected by intracellular Cl^-^ and pH changes (Funke et al., 2011), which is crucial in view of the massive ionic disturbances during metabolic compromise and SD. With these properties it outperforms any synthetic dye such as the fluorescein and rhodamine-derivatives used earlier to detect ROS formation and oxidative stress [see for example (Foster et al., 2006; Can et al., 2017)]. Furthermore, our current experiments exclude any interferences with repeated illumination, DMSO, and autofluorescence of vital tissue (Spitzer et al., 2011). Nevertheless, as demonstrated by isosbestic point-excitation, roGFPc fluorescence is affected (decreased) by the altered optical properties in the SD-invaded tissue. SD is associated with an intrinsic optical signal (IOS), which represents cell volume and morphological changes as well as changes in tissue autofluorescence within the invaded tissue (Andrew et al., 1999; Müller and Somjen, 1999; Müller and Mané, 2010; Sword et al., 2024). This IOS is typically measured as light reflectance, light transmission or as tissue autofluorescence. As demonstrated in Figure 1H, tissue autofluorescence is too weak to markedly contribute in our experiments. Reflected or transmitted light are of no concern as any backscattered excitation (395 and 470 nms) is blocked by the 495 dichroic and 525/50 emission bandpass filter (see Figure 1A) from reaching the CCD camera. So what remains as a potential contribution under our recordings conditions are cell volume changes arising from the SD-related cell swelling. These can, however, be considered to be minimized by the ratiometric approach applied here for roGFP-based redox imaging.

### 4.2 Spreading depolarization-mediated neuronal oxidation

Parallel recordings of extracellular DC potentials and neuronal redox conditions confirmed a pronounced roGFPc oxidation during SD. This applied to all types of SD induction tested, i.e., O_2_ withdrawal, K^+^ microinjection and mitochondrial uncoupling. The oxidation of roGFPc was strictly linked to the electrical signs of SD, starting together with the ΔV_o_ but then building up more slowly. It represents an oxidizing shift in cytosolic thiol redox balance due to intensified cellular ROS production overwhelming the cell-endogenous redox-buffering systems. The very oxidant species underlying these changes, cannot be specified for sure. Based on the reactivity and lifetimes of various oxidants, most of their cellular signaling is considered to arise from H_2_O_2_, as it shows the longest lifetime and hence may diffuse appreciable distances within cells (Pryor, 1986). This view is also supported by the intensified H_2_O_2_ levels detected upon K^+^-induced SD in rat cortex (Viggiano et al., 2011).

Earlier studies taking advantage of lucigenin chemiluminescence to monitor the time course of superoxide formation in rat cortex *in vivo* obtained a somewhat different picture: K^+^-induced cortical spreading depression did not induce any detectable superoxide formation, whereas cortical spreading ischemia or transient global ischemia led to a decline in superoxide levels during the ischemic phase, followed by increased superoxide formation during reperfusion (Dirnagl et al., 1995; Dreier et al., 1998). Peri-infarct depolarizations arising during the ischemic period did not affect lucigenin chemiluminescence (Peters et al., 1998). Applying this approach to corticohippocampal brain slices, showed a similar biphasic pattern of dampened superoxide formation during hypoxia and an oxidative burst upon reoxygenation (Dirnagl et al., 1995). To understand these differences, the detailed properties of the two sensors need to be considered. Lucigenin chemiluminescence, a probe for superoxide (Li et al., 1998), reports the very time point of superoxide production, but not the resulting consequences for the cellular/tissue environment. It lacks, however, cellular/compartmental specificity, requires complex reaction pathways, and its reliability has been debated (Faulkner and Fridovich, 1993; Liochev and Fridovich, 1998). In contrast, roGFP is fully embedded into cellular redox homeostasis, reporting in a catalyzed reaction cell/compartment specific changes in thiol (especially glutathione) redox balance (Dooley et al., 2004; Björnberg et al., 2006). The roGFP1 sensor used here, is not specific to a particular type of ROS, but reports the consequences of altered ROS production and/or altered cellular scavenging capacities (Hanson et al., 2004; Meyer and Dick, 2010). Accordingly, an oxidizing shift of cellular thiol redox balance may not only arise from an increased generation of ROS, but also from an exhausted cell-endogenous antioxidant capacity. This may include the failure to reinstate the reduced forms of cellular redox couples, for example during shortage of the master electron donor NADPH, with accumulating oxidized redox couples potentially acting as pro-oxidants (Dey et al., 2016). So, whereas the superoxide burst upon reoxygenation/reperfusion (Dirnagl et al., 1995; Dreier et al., 1998) can be assumed to correspond to the late phase of roGFP oxidation and its secondary further increase, more work will be required to decipher in detail, how the observed drop in lucigenin chemiluminescence during the hypoxic/ischemic phase can be connected to the initial roGFP oxidation we observed in neuronal cytosol and mitochondrial matrix. Along this line, it also remains to be clarified, why lucigenin chemifluorescence does not show superoxide production during normoxic SD, whereas we observed a clear roGFPc oxidation, and others showed increased H_2_O_2_ levels, intensified superoxide dismutase activity and lipid peroxidation, as well as ROS formation detected as CellROX fluorescence under these conditions (Viggiano et al., 2011; Grinberg et al., 2012; Shatillo et al., 2013).

The very extent of roGFPc oxidation depended on the type of SD, and was most intense during HSD. O_2_ deprivation tremendously disturbs cell metabolism (Kass and Lipton, 1982; Lipton and Whittingham, 1982; Whittingham et al., 1984). Accordingly, mitochondria are under extreme stress and at least initially attempt to compensate for a fading O_2_ supply with intensified activity, in particular as mitochondrial respiration is capable of operating down to very low O_2_ levels (Mills and Jöbsis, 1972; Gnaiger et al., 1995). In contrast, K^+^ microinjection and the resulting normoxic SD primarily disrupt ionic homeostasis and challenge cell metabolism, but do not shut it down as hypoxia eventually does. Furthermore, normoxic SD mediates a temporary challenge as – in contrast to HSD – it is self-limiting (Lipton, 1999; Somjen, 2001). Overall, this obviously resulted in a less severe impact on neuron function and an attenuated ROS formation.

When mitochondria were uncoupled, FCCP administration swiftly mediated a reducing shift. The uncoupler FCCP dissipates the mitochondrial membrane potential (ΔΨm) by transporting protons across the inner mitochondrial membrane (Benz and McLaughlin, 1983). As mitochondria-based ROS formation depends on an intact ΔΨm (Abramov et al., 2007), uncoupling dampens their ROS output. Accordingly, the FCCP-mediated reducing shift can be assumed to represent the constant ROS load normally imposed onto cellular redox balance by functional mitochondria. The fact that the FCCP-induced reducing shift appeared quite large in comparison to the SD-mediated oxidation likely arises from the fact that cellular redox balance efficiently buffers especially oxidizing stimuli. Also, as roGFP oxidation competes with the cellular ROS-scavenging systems, the extent of SD-related oxidation might have been under-estimated. In future studies it may therefore be of interest to assess to what extent the SD-mediated oxidation is boosted by, e.g., catalase inhibitors.

The time to onset of the FCCP-induced SD was markedly longer as compared to O_2_ withdrawal. This likely reflects the gradual tissue penetration of FCCP and slower time-scale at which mitochondrial inhibition and the resulting cellular ATP depletion occur. The roGFP oxidation during chemically-induced SD was less intense than during HSD, but comparable to normoxic SD. This dampened oxidation during FCCP-induced SD obviously reflects the limited availability of mitochondria-derived ROS. Yet it also proves, that despite mitochondrial uncoupling substantial amounts of ROS were still produced, which argues against mitochondria being the major ROS generator during SD. This is also suggested by the pilot experiments with mitochondria-directed roGFPm, which revealed only a moderate oxidation of mitochondrial matrix during HSD. Therefore, most of the ROS underlying the observed roGFPc oxidation is apparently not of mitochondrial origin.

A unique feature of the K^+^-induced SDs was the reversibility of the roGFPc oxidation in ~40% of the slices. K^+^ efficiently triggered SD, but did not massively impair cellular/mitochondrial metabolism, thereby mediating a less severe impact. The variable degree of reversibility observed for normoxic SD obviously arose from the actual amounts of K^+^ microinjected for SD induction, as too high K^+^ levels may provoke terminal, non-reversible SDs (Herreras and Somjen, 1993b; Dreier, 2011). In contrast, the regenerative capacity of the tissue after SD was rather limited during hypoxia-and FCCP-induced SDs. Even though the ΔV_o_ eventually ceased, roGFPc oxidation did not and even continued slowly. As proven by recording fEPSPs during HSD, a recovery of synaptic function was not evident under these conditions. In part, this may arise from the delayed reoxygenation (3 min upon SD onset) and the submerged condition. Furthermore, Na_2_SO_3_ and FCCP were probably not washed out sufficiently fast to fully maintain tissue viability.

During hypoxia, a slight oxidative shift already occurred before HSD onset. Whereas this pre-SD oxidation remained unaffected by DPI, allopurinol or Ca^2+^ withdrawal, it was not evident, during K^+^- or FCCP-induced SDs. Imaging of roGFPm revealed a moderate matrix oxidation before HSD onset, which identifies mitochondria as the responsible ROS generator during early hypoxia (Abramov et al., 2007). This initial oxidation coincides with the early K^+^ release into the interstitial space (Hansen, 1978; Hansen and Zeuthen, 1981), which increasingly challenges the resting membrane potential, intensifies ATP consumption, and stimulates mitochondrial respiration – at least as long as any remaining O_2_ is still available and mitochondrial function has not collapsed.

The observed shapes of the SD-related DC shifts were quite variable (see Figures 2A–D). As observed earlier in interfaced rat hippocampal slices, especially FCCP-induced SDs showed very short durations of the DC potential deflection (Gerich et al., 2006). Other sources of this variability even for identical SD-inducing stimuli may be the electrode position relative to the SD induction site during K^+^ microinjection as well as the exact tissue insertion depth of the recording electrode. In contrast, for a given mode of SD induction, the temporal profiles of roGFPc oxidation were less variable, which may be due to their slower kinetics. For a detailed correlation of the electrophysiological SD parameters and roGFPc oxidation properties, a larger number of experiments and also increased imaging frame rates should be considered. In such future experiments, intracellular membrane potential recordings might then provide more detailed insights than the currently monitored extracellular DC potentials.

### 4.3 Wavefront propagation of roGFPc oxidation

The low optical magnification applied unveiled the wavefront propagation of the SD-associated redox changes. The oxidative wavefront propagated at 2.61 ± 0.41 mm/min during HSD, at similar velocity during K^+^-induced SD, but it was slower for FCCP-induced SDs (2.11 ± 0.47 mm/min). Thus, for the propagation of the redox alterations, it is not relevant whether the oxidation is linked to a normoxic or an hypoxic SD. Yet, a clear dependence on extracellular Ca^2+^-availability was observed. Therefore, the extent of neuronal Ca^2+^ influx is not only a key factor for the amount of ROS produced during SD, but it also facilitates the oxidative impact on the surrounding tissue by promoting SD propagation. This is in line with the pivotal role of Ca^2+^ influx for the propagation of especially normoxic, K^+^-induced SD (Jing et al., 1993; Dietz et al., 2009).

### 4.4 Reactive oxygen species generating sources during SD

As key event for SD-related ROS formation, we identified neuronal Ca^2+^ influx from extracellular space and the resulting massive cellular Ca^2+^ load. Withdrawing extracellular Ca^2+^ did not affect baseline redox balance, but the magnitude of the SD-related roGFPc oxidation and the wavefront propagation velocity were significantly decreased during normoxic SD as well as hypoxia-induced SD. Furthermore, in the absence of extracellular Ca^2+^, the roGFPc oxidation mediated by HSD and K^+^-induced SD gained partial or even full reversibility, even though the ΔV_o_ remained unchanged. In line with earlier studies, it is not the mere depolarization or hypoxia *per se* which causes potential cell damage. If extracellular Ca^2+^ is withdrawn before O_2_ deprivation or if Ca^2+^ influx is prevented during HSD, neurons can fully recover their function even after a usually damaging hypoxia period (Kass and Lipton, 1982; Balestrino and Somjen, 1986). However, as seen also in the present experiments, lack of extracellular Ca^2+^ does not prevent the occurrence of normoxic or hypoxic SD. Furthermore, mitochondria still depolarize during SD in Ca^2+^-free solutions (Bahar et al., 2000); hence the protection mediated by these conditions cannot be based on preventing a loss of ΔΨm. Moreover, not Ca^2+^ *per se* provokes neuronal damage. HSD, immediately followed by reoxygenation, is reversible despite massive cellular Ca^2+^ influx. To cause cell damage, neuronal Ca^2+^ load must be maintained for a critical period (Siesjö, 1986), which then certainly also provokes a Ca^2+^-driven ROS generation by mitochondria (Duchen, 2000; Adam-Vizi and Starkov, 2010). Under the conditions of anoxia/reoxygenation, however, Ca^2+^ was reported to activate also NADPH oxidase (Abramov et al., 2007). Furthermore, pronounced cellular Ca^2+^ load may convert xanthine dehydrogenase into the superoxide-producing xanthine oxidase (Dykens et al., 1987; Cheng and Sun, 1994). The longer such strongly oxidative conditions persist, the more serious are the consequences for the affected neurons, as ROS overproduction and the oxidizing milieu massively disturb cellular redox homeostasis, leading to transient or permanent damage of cell components and ultimately cell death (Valko et al., 2007).

In addition to HSD, also normoxic SD mediates a pronounced cellular Ca^2+^ influx, but does not result in permanent neuronal damage (Nedergaard and Hansen, 1988; Herreras and Somjen, 1993a). Time is the key factor. Normoxic SD is usually self-limiting and therefore the duration of cellular Ca^2+^ load not sufficient to provoke lasting cell damage. In addition, tissue recovery from normoxic SD and reinstation of normal ion distribution is facilitated by a less severely impaired cellular metabolism and still sufficient ATP availability. A persistent normoxic SD, however, is irreversible and results in a loss of neuronal viability (Kawasaki et al., 1988).

In hippocampal neurons, an active NADPH oxidase contributes to cell-endogenous ROS production (Tejada-Simon et al., 2005). Furthermore, it represents a main source of NMDA receptor-mediated ROS generation (Brennan et al., 2009). During anoxia/reoxygenation, NADPH oxidase seems to be activated by a rise in intracellular Ca^2+^ (Abramov et al., 2007). A similar Ca^2+^-dependence may be suggested by our observation that roGFPc oxidation was dampened and gained reversibility when SD was evoked in Ca^2+^-free conditions. Pharmacological intervention with the NADPH oxidase inhibitor DPI and the xanthine oxidase inhibitor allopurinol, however, mediated only minor effects on SD and roGFP oxidation. Upon allopurinol treatment the intensity of the HSD-associated roGFPc oxidation tended to decrease, which may indicate some contribution of xanthine oxidase. In contrast, DPI intensified the roGFPc oxidation during normoxic SD and shortened the ΔV_o_ duration, which suggests an improved synchronization of the neuronal/glial depolarization and hence an intensified SD. In line with this, the reversibility of the roGFPc oxidation became limited when normoxic SD was induced in the presence of DPI. As DPI may also interfere with complex I of the mitochondrial respiratory chain (Holland et al., 1973), it might have partly impaired mitochondrial metabolism, thereby limiting ATP production and thus the capacity to recover from the normoxic SD. Under hypoxic conditions this was not evident, as mitochondrial metabolism was blocked already by the lack of O_2_. In support of the assumed side effect of DPI on mitochondrial performance, we earlier observed in rat hippocampal slices that the onset of HSD was hastened when slices were pre-treated with DPI (Gerich et al., 2006).

In AO-treated mice, only a slight increase in the ΔV_o_ amplitude during HSD was seen, other HSD parameters, normoxic SD or the associated roGFPc oxidations remained unaffected. As we showed earlier, the AO feeding moderately increased the body weights of 7 weeks old male wildtype mice, and slightly reduced their brain weights (Baroncelli et al., 2022). Screening for a stabilization of mitochondrial function in AO-treated female mice (50 days old), we observed an increase in citric synthase activity in cortex and brainstem, but not in hippocampus, cerebellum and midbrain, which may suggest brain region-specific merits of this treatment (L. van Agen and M. Müller, unpublished). In the current study, in view of cellular redox balance only a tendency toward more reducing steady-state baseline conditions was found in the AO-fed male mice. It therefore seems that for a more successful modulation of SD and neuronal redox conditions in hippocampus, the composition of the AO cocktail may need further optimization. Others reported that ascorbic acid (vitamin C) treatment of rats decreased the propagation velocity of K^+^-induced cortical SD at low concentrations, whereas higher concentrations increased the propagation velocity (Mendes-da-Silva et al., 2014).

An interesting aspect is that the roGFPm oxidation in mitochondrial matrix during HSD was markedly less intense than the cytosolic redox changes, showed a faster time course and fully recovered upon reoxygenation. In part, this may be due to the fact that mitochondria are more efficiently redox buffered than, e.g., the cytosol and that they are capable of decomposing ROS largely independent of cytosolic antioxidant pathways (Dey et al., 2016). No such recovery upon HSD was seen in the cytosol. Therefore, the secondary slow continuous oxidation observed with roGFPm as well, may in part represent an entry of cytosol-derived ROS into mitochondrial matrix. Accordingly, it may be the cytosolic redox conditions that contribute to the delayed mitochondrial dysfunction. Of course not all mitochondria-generated ROS enter mitochondrial matrix, some are directly diverted to the mitochondrial intermembrane space from where they may escape into the cytosol (Brand, 2010). Nevertheless, the roGFPm imaging supports the notion that mitochondria are not the main source of SD-related ROS formation.

### 4.5 Outlook

Based on the performed first-time assessment of redox dynamics, hypoxia-, FCCP-and K^+^-mediated SDs were clearly associated with a marked oxidation of the neuronal cytosol. Mitochondria are clearly not the sole contributor, but in concert with non-mitochondrial sources generate ROS in a Ca^2+^-dependent manner. The very routes of Ca^2+^ entry and further potentially contributing cellular ROS sources still have to be defined in subsequent studies, in particular as a different set of mechanisms may be involved for chemically-, hypoxia-and K^+^-induced SDs. Furthermore, exhaustion of cellular antioxidant systems should be considered as a potential component contributing to roGFP oxidation during and upon SD. Also in later studies higher frame rates might be advisable to improve time resolution and to characterize the spatial propagation of neuronal redox alterations more closely. Ideally, redox imaging could be attempted also at higher resolution on the single-cell level, but in view of the massive ion changes and the resulting tissue swelling during SD this would then require technical approaches to stabilize the optical focal plane.

## Data Availability

The raw data supporting the conclusions of this article will be made available by the authors, without undue reservation.
